# Noble-metal-free hydroxyapatite activated by facile mechanochemical treatment towards highly-efficient catalytic oxidation of volatile organic compound

**DOI:** 10.1038/s41598-021-86992-8

**Published:** 2021-04-05

**Authors:** Yunzi Xin, Takashi Shirai

**Affiliations:** 1 Advanced Ceramics Research Center, Nagoya Institute of Technology, Nagoya, 466-8555 Japan; 2 Department of Life Science and Applied Chemistry, Graduate School of Engineering, Nagoya Institute of Technology, Nagoya, 466-8555 Japan

**Keywords:** Environmental sciences, Materials for energy and catalysis

## Abstract

Controlling of volatile organic compound (VOC) emitted from industrial processes as most abundant and harmful air pollutant, has become one of the most important global environmental issues due to the rapid urbanization and industrialization. As an alternative and new type catalyst instead of conventional noble-metal nanoparticles widely utilized in oxidative decomposition of VOC, here we report the superior catalytic performance with 100% CO_2_/CO conversion on hydroxyapatite (HAp, Ca_10_(PO_4_)_6_(OH)_2_) with structurally well-controlled active surface tailored via facile one-step mechanochemical treatment in ambient air. With detailed characterizations of particle morphology, crystallinity and chemical structure with respects to surface defect/oxygen vacancy formation, acidity/basicity and VOC affinity on HAps activated through different mechanical stresses when altered ball size is utilized in planetary ball-milling assisted mechanochemical process, it was found that the predominant defect/oxygen vacancy generation in PO_4_^3−^ site and enhanced basic site population established by selective mechanochemical activation of c-plane, facilitates the favorable catalytic oxidation route towards highly-efficient CO_2_/CO conversion of VOC. Regards to the cost-effectiveness and non-toxic nature of HAp, incorporated with the sustainable mechanochemical surface structure tuning process, the results presented in this work opens new strategy in development of novel noble-metal-free catalyst for VOC elimination and environmental cleaning techniques.

## Introduction

Volatile organic compound (VOC) emitted from industrial processes including chemical manufacturing, printings, power plants and automobiles, is one of the most abundant and harmful air pollutants as precursors of photochemical smog and the secondary aerosol. VOCs also bring extremely harmful impact on human beings due to their toxic and carcinogenic nature^1–3^. The controlling of VOC emission has become one of the most important global environmental issues due to the rapid urbanization and industrialization. Conventionally, VOC controlling techniques involving condensation^4^, adsorption^5^, biological degradation^6^, and catalytic oxidation^7–15^ have been established. Catalytic oxidation via thermal-/photo- or plasma-assisted processes has attracted much attention owing to the possibility of conversing VOC into harmless CO_2_ and H_2_O as final product. Among the above all, thermal catalytic oxidation via noble-metal (as platinum, palladium, gold, silver, etc.) nanoparticle supported on porous ceramic carrier has been numerously reported^8–12^. However, regards to the high-cost, critical requirement on nanoparticle size and dispersibility controlling, development of alternative and novel noble-metal-free catalyst is urgently desired. Supported transition metal and/or transition metal oxides as cheaper alternative to noble-metal catalysts, possess several advantages such as excellent thermal stability and resistance to poisoning^9,16^. Perez et al. reported the oxidative combustion of VOC on Mn-SBA15 catalysts synthesized by utilizing different Mn precursors^17^. Dou’s group was succeeded in achieving efficient catalytic removal of ethyl acetate over CuCe_x_Zr_1-x_O_y_/ZSM-5 compounds by adjusting the chemical composition (x = 0, 0.25, 0.5, 0.75, 1)^18^. As examples of metal oxide case, oxidation of VOCs over lattice doped CoO_x_-CeO_2_ and MnO_x_-CeO_2_ are reported during where the chemical state of dopant ion in altered x value and its influence on catalytic activity are systemically investigated^19,20^. In addition, perovskite catalyst is also widely studied towards VOC combustion. Pecchi et al. reported the catalytic combustion of ethanol and acetyl acetate on LaFeO_3_, LaNiO_3_ and substituted LaFe_1-y_Ni_y_O_3_ perovskites^21^. They mentioned the substitution degree of Ni with corresponding modulation of oxygen adsorption and release plays an important role in deciding catalytic activity. However, the indispensable requirement in synthesis of complex molecular as well as precise chemical composition controlling is the main drawback of above catalysts.

Hydroxyapatite (HAp, Ca_10_(PO_4_)_6_(OH)_2_), as one of the most well-known calcium phosphate materials, has attracted worldwide interests in variable applications as bio-ceramics for artificial bone tissue crafting, adsorbents of proteins and ion-exchanging fillers for heavy-metal ions^22–24^. With the discovering of active radical generated via reaction between adsorbed O_2_ molecular with trapped electron induced through thermal dehydration of surface hydroxyl group, we previously reported the complete catalytic decomposition of VOC on HAp and initially derived the catalytic reaction mechanism which also emphasizes the important role of the co-existed acidic/basic sites in HAp^25–27^. Such results open a new possibility for designing of noble-metal-free catalysts for VOC control and environmental cleaning technologies due to the cost-effectiveness and super facile synthetic approaches of HAp. Although complete decomposition of ethyl acetate is obtained previously, a relative low CO_2_ selectivity with existence of organic substance as secondary VOC pollutant is also confirmed in final product^25^. Regards to the fact that the reaction route in catalytic oxidation of VOC significantly depends on the chemical structure of HAp^25–27^, herein HAp with structurally well-controlled active surface is tailored via one-step mechanochemical treatment in ambient air, designed for pursuing highly-efficient catalytic oxidation of VOC and a 100% CO_2_/CO conversion with zero residual of organic substance. The morphology, crystallinity and chemical structure with respects to surface defect/oxygen vacancy formation, acidity/basicity and VOC affinity of HAps that activated through different mechanical stresses when altered ball size is utilized in planetary ball-milling assisted mechanochemical process, were investigated systemically with characterizations assisted by scanning electron microscopy, powder X-ray diffraction, Fourier transform infrared spectrometer, X-ray photoelectron spectroscopy, in-situ electron spin resonance analysis, surface acidity/basicity evaluation, in-situ gas-flowing diffuse reflectance infrared Fourier transform spectroscopy. Merge with catalytic performance test on oxidative decomposition of VOC, the correlation between surface chemical structure of mechanochemically activated HAp and catalytic activity for oxidative decomposition of VOC is also clarified. It was found that the predominant defect/oxygen vacancy generation in PO_4_^3−^ site and enhanced basic site population established by selective mechanochemical activation of c-plane, facilitates the favorable catalytic oxidation route towards highly-efficient CO_2_/CO conversion of VOC. Regards to the cost-effectiveness and non-toxic nature of HAp, incorporated with the sustainable mechanochemical surface structure tuning process, the results presented in this work opens new strategy in development of novel noble-metal-free catalyst for VOC elimination and environmental cleaning techniques.

## Results and discussion

**Figure 1:**
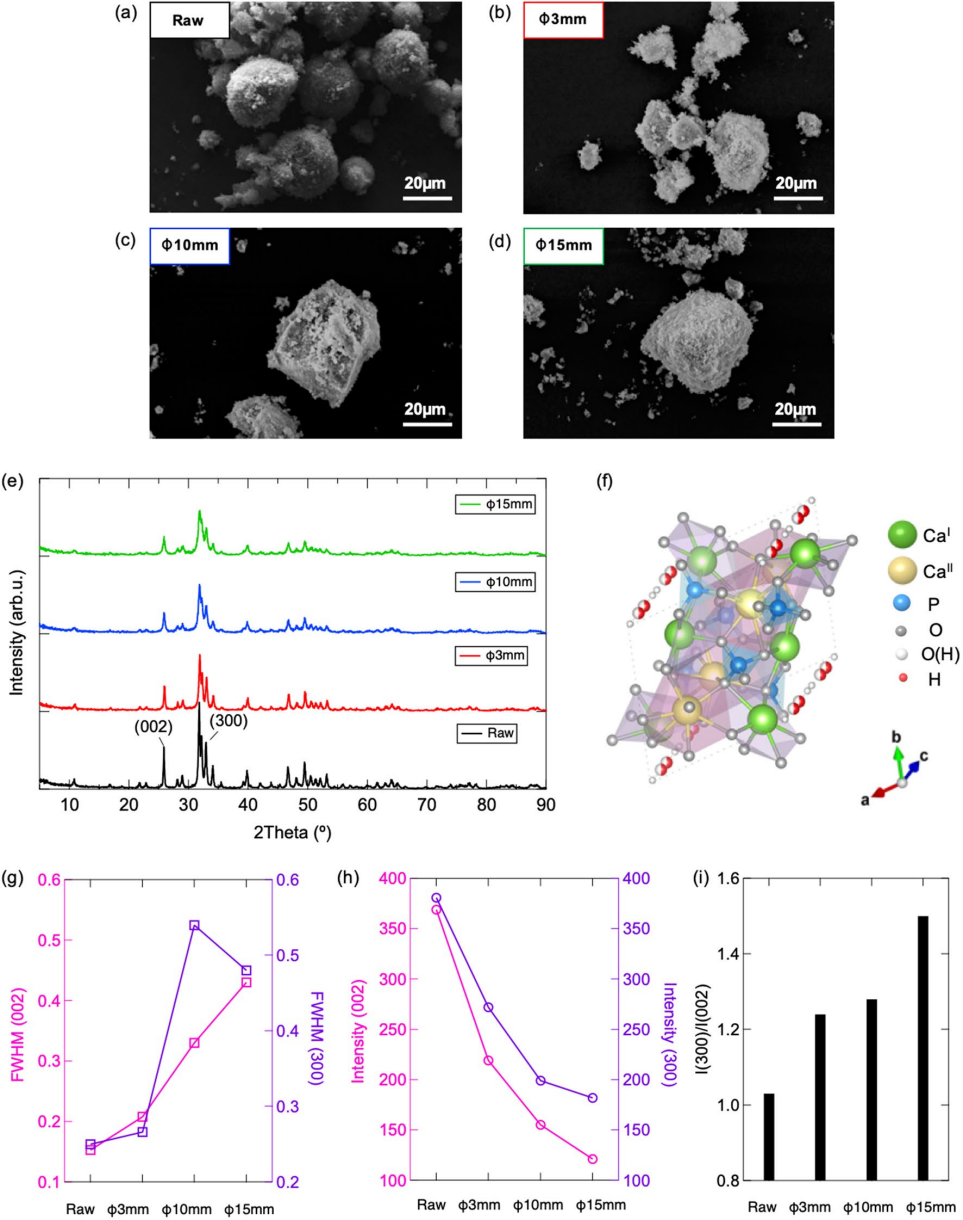
(**a**–**d**) SEM images and (**e**) PXRD patterns of raw and mechanochemically treated HAps; (**f**) hexagonal crystal structure of stoichiometric HAp (*P*63/m: [Ca^I^_4_Ca^II^_6_](PO_4_)_6_(OH)_2_); (**g**) full width half maximum (FWHM), (**h**) peak intensity and (**i**) intensity ratio of (002) and (300) planes analyzed from PXRD patterns.

Figure 1 demonstrates the morphology of raw and mechanochemically treated HAp observed by SEM. Sphere agglomerate with average diameter of 20 µm, which consists of nanoscale primary particle is observed in raw HAp. Compare with the loose-packing of primary particles in raw HAp, agglomerates of close-packed particles are confirmed in mechanochemically treated samples. Rare change in particle morphology is obtained in 3 mm ball treated HAp, while the size of agglomerate grows obviously as the ball diameter increases to 10 and 15 mm. It can be suggested that more mechanical stress was induced via 10 mm and 15 mm balls in planetary ball-milling process, which results in grinding and re-aggregation of primary particle. In addition, the BET sufficient surface areas of raw, 3 mm, 10 mm and 15 mm ball treated HAp powders are estimated as 40.409, 32.577, 22.548 and 16.630 m^2^/g respectively, whose results also see good agreement with the results obtained from SEM images.

**Figure 2:**
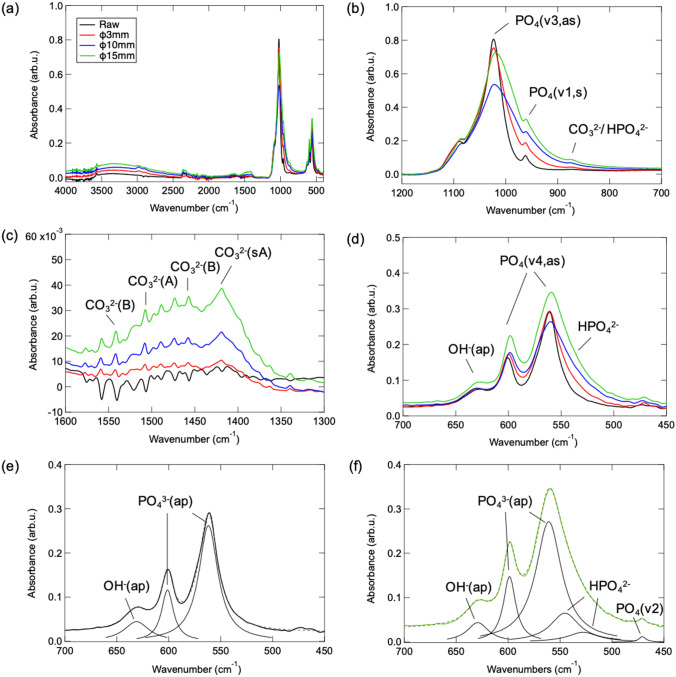
ATR-FT/IR spectra of raw and mechanochemical treated HAp (**a**) in entire wavenumber region, partially expended (**b**) PO_4_^3−^ region, (**c**) CO_3_^2−^ region, (**d**) HPO_4_^2−^ region and corresponding fitting results of (**e**) raw and (**f**) mechanochemical treated HAp.

The crystal structure of raw HAp and mechanochemically treated samples is characterized via PXRD and the corresponding results are given by Fig. 2. Mono-phase of HAp with hexagonal crystal structure (*P*63/m, drawn as Fig. 1f) is confirmed in each spectrum without appear of any other crystal phases and impurity contains. Regards to the increase of full width half maximum (FWHM) and decrease of peak intensity of (002) and (300) planes as summarized in Fig. 1g, h, it demonstrates that the structural disordering is induced in crystal structure of HAp after mechanochemical treatment, which is probably cause by the generation of surface defects via mechanical energy^28^. Interestingly, an increase of peak intensity ratio of (300)/(002) planes is also confirmed as Fig. 1i, which suggests that the surface tailoring originated through mechanochemical treatment tends to progress predominantly and selectively on c-plane than a-plane.

**Figure 3:**
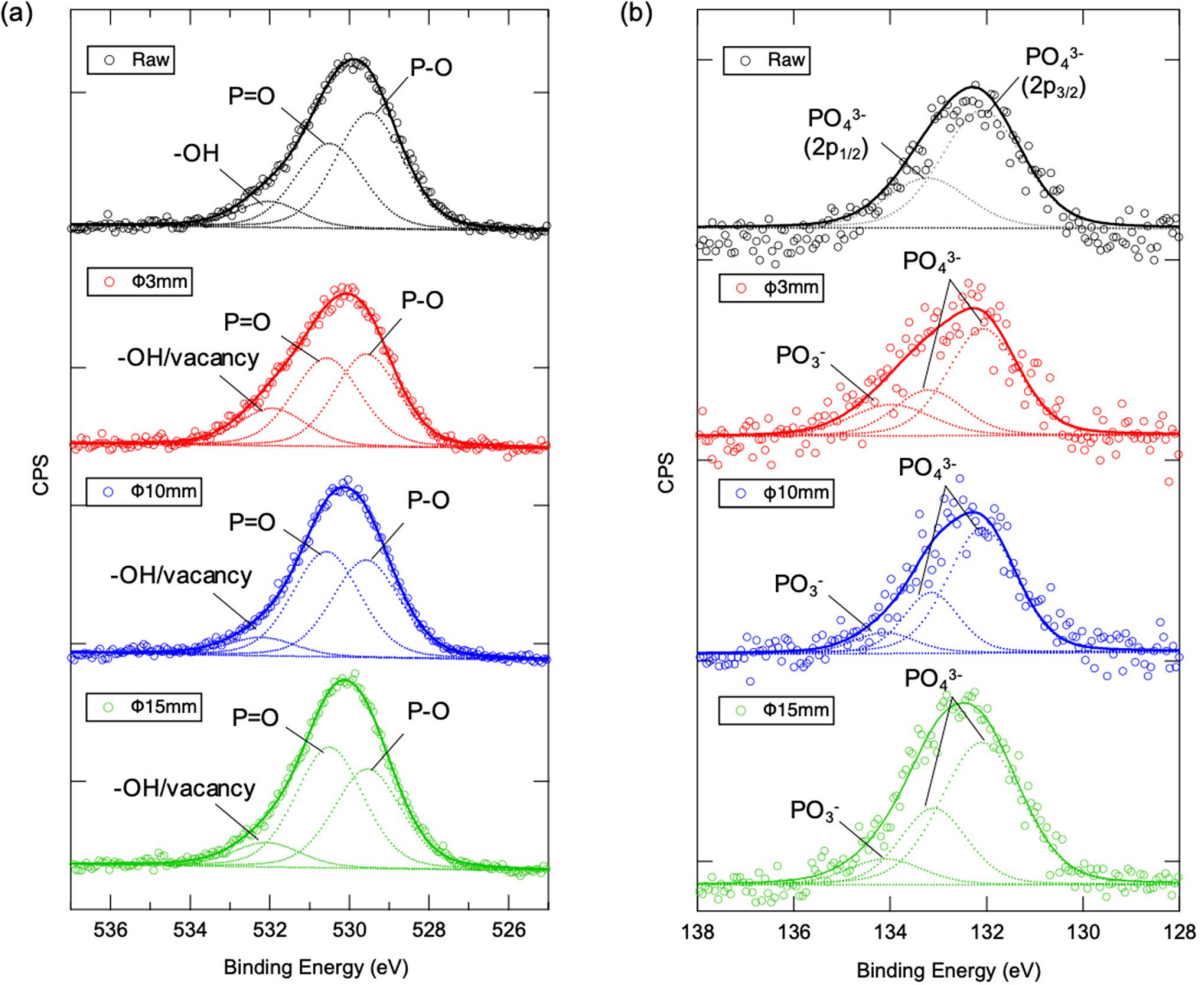
XPS spectra of (**a**) O1s and (**b**) P2p orbitals of raw and mechanochemically treated HAps.

The chemical structure of HAps before and after mechanochemical treatment are further analyzed in details via ATR-FT/IR characterization and the observed spectra in entire and partially expended wavenumber regions are displayed in Fig. 2. As observed spectra in entire wavenumber in Fig. 2a, stretching mode of typical apatitic hydroxyl group is confirmed at 3570 cm^−1^ in all samples^29–31^. Regards to the partially expended spectra of PO_4_^3−^ site represented in Fig. 2b, peaks around 1023 and 962 cm^−1^ are assigned to the asymmetric and symmetric stretching vibration mode of P–O and P=O respectively, while shoulder component appears at 870 cm^−1^ probably belongs to substituted CO_3_^2−^ and/or HPO_4_^2−^ ^32–34^. By comparing the mechanochemically treated HAp with raw one, it can be illustrated that asymmetric stretching decreases as mechanical stress induces. Moreover, the peak top of P–O asymmetric stretching shifts to smaller wavenumber region accompanying with enlarged peak width, which also results in a reversed increase tendency of peak intensity in 15 mm ball case. It has been reported that the protonation of PO_4_^3−^ and formation of HPO_4_^2−^ produce symmetry reduction from tetrahedral T_*d*_ to C_3ν_, which causes splitting of 1023 cm^−1^ (ν3, as) into two separated side components (ex. 1011 splits to 1078 and 990 cm^−1^ in reference^35,36^). Thus, we suggest the formation of HPO_4_^2−^ accompanied peak splitting contributes to the enlarged peak width, and abundant HPO_4_^2−^ existed in HAp treated with the strongest mechanical stress (15 mm ball) probably attributes to the significant intensity increase of splitting peak at smaller wavenumber side, to show a reversed peak intensity increase and shift. Figure 2c illustrates the spectra focusing on the multiple peaks observed around 1400–1570 cm^−1^, which originate from the asymmetric stretching mode of substituted CO_3_^2−^ ^37,38^. Peaks appear at 1507 and 1420 cm^−1^ are attributed to bulk and surface type A carbonate substituted on OH^−^ site. On the other hand, peaks at 1545 and 1456 cm^−1^ are assigned to bulk type B carbonate corresponding to substitution through PO_4_^3−^ site^32^. It can be concluded that both A- and B-type CO_3_^2−^ substitutions occur in mechanochemically treated HAps, and the substitution degree increases as mechanical energy enhances when the size of ball utilized in planetary ball-milling increases. Figure 2d displays fingerprint region involving peaks of asymmetric bending mode of P–O at 565 and 605 cm^−1^, as well as liberational band of OH^−^ at 631 cm^−1^. In addition, it is obvious that a shoulder peak appears around 530 cm^−1^ after mechanochemical treatment. Such component is further investigated via peak-fitting analysis for raw and 15 mm-ball mechanochemical treated HAp. As results shown in Fig. 2e, f, characteristic peaks of HPO_4_^2−^ at 530 and 550 cm^−1^ ^39,40^ appear clearly in mechanochemically treated HAps. By summarizing the above detailed results observed in ATR-FT/IR spectra of raw and mechanochemically treated HAps, it can be deduced: (a) Mechanochemical process induces surface defect/oxygen vacancy generation predominantly on PO_4_^3−^ site especially in part of asymmetric bonding of P–O, whose result shows good agreement with the fact that surface tailoring via mechanochemical treatment selectively promotes principally and selectively on c-plane where PO_4_^3−^ ions exist (see Fig. 1 and related description). (b) Substitution of CO_3_^2−^ ions takes place both on PO_4_^3−^ and OH^−^ sites during mechanochemical treatment which might be caused by the existence of CO_2_ gas encapsulated in vessel of planetary ball-milling system during sample loading in an ambient air atmosphere. (c) HPO_4_^2−^ can be formed in mechanochemical treatment, which must be corresponding to the reaction between HAp and water existed in ambient air. In addition, it can be also exemplified that surface defect/oxygen vacancy generation, substitution of CO_3_^2−^ ions and formation of HPO_4_^2−^ is stimulated gradually when the higher mechanical stress energy is created through planetary ball-milling with larger balls. The chemical structure change of HAp after mechanochemical treatment is also further investigated via XPS characterization. As results shown in Fig. 3a, the XPS spectra of O1s orbital of raw HAp consists of three species at 529.50, 530.50 and 532.03 eV assigned to P–O, P=O and hydroxyl oxygen –OH^41,42^. The relative composition of P–O decreases as the strengthened mechanical stress induces by larger ball in planetary ball-milling process, which exemplifies mechanochemical treatment selectively promotes surface defect generation on PO_4_^3−^ site especially in part of asymmetric bonding of P–O. Moreover, the peak intensity at 531.99 eV increases in the case of HAp mechanochemically treated via 3 mm ball, whose phenomenon can be attributed to the generation of oxygen vacancy with the fact that corresponding characteristic peak will also emerge in such higher energy region^42,43^. In the case of P2p orbital in Fig. 3b, spectra of raw HAp can be separated into bi-components at 133.23 and 132.10 eV due to spin-orbital splitting of 2p_1/2_ and 2p_3/2_, originates from P^5+^ tetrahedrally coordinated to O^41–44^. On the other hand, peak component at higher binding energy apart ~ 1.70 eV from PO_4_^3−^, corresponding to defective PO_3_^−^ appears in mechanochemical treated HAp^45^. Corresponded well with the results of ATR-FT/IR, it can be concluded that mechanochemical process induces surface defect/oxygen vacancy predominantly on PO_4_^3−^ site. Thus, it can be supposed that a new surface with selectively well-controlled chemical structure on HAp is tailored through the facile one-step mechanochemical treatment.

**Figure 4:**
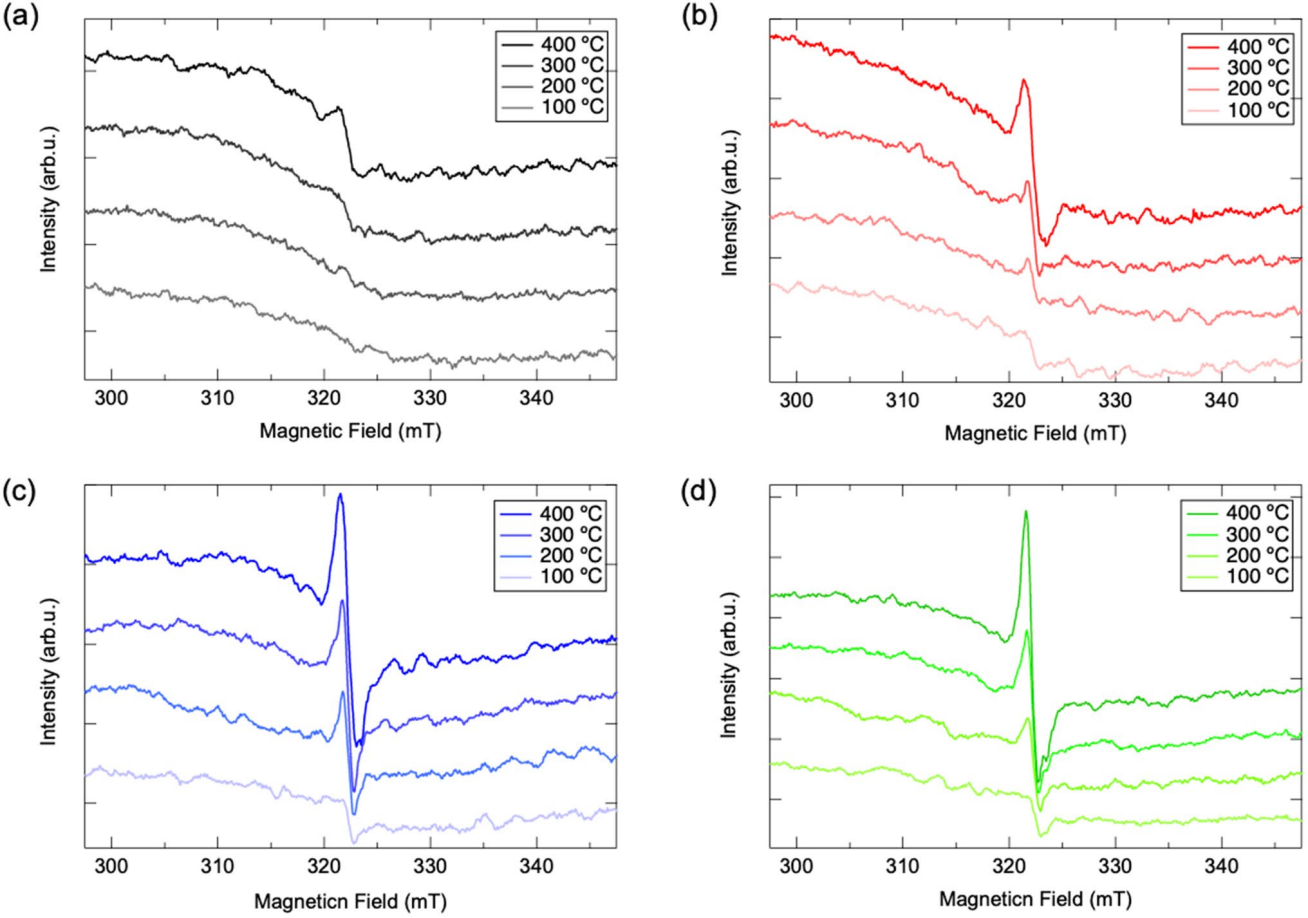
In-situ ESR spectra of (**a**) raw and mechanochemical treated HAp via (**b**) 3 mm, (**c**) 10 mm and (**d**) 15 mm under different temperatures.

To evaluate the surface defect/oxygen vacancy produced in mechanochemical treated HAp and corresponding structure change under thermal activation, ESR analysis with in-situ heating is conducted on raw and mechanochemically treated HAps. As results demonstrated in Fig. 4a, signal at g = 2.002 is observed in raw HAp when temperature is getting higher than 200 °C. Such signal has been assigned as trapped electron induced on HAp through dehydration of surface hydroxyl group, which is responsible for the generation of super active O_2_ radical through reaction with surface adsorbed O_2_ molecular^25–27^. Regards to the spectra given by Fig. 4b–d, such peak with significant higher spin density and sharper signal is detected in mechanochemically treated HAps even at lower temperature of 200 °C. Regards to the above XRD, ATR-FT/IR and XPS results, the intensified signal can be attributed to the large number of trapped electrons on surface defects/oxygen vacancies produced in c-plane existed PO_4_^3−^ sites through mechanochemical treatment, wherein the density of radicals also gradually increases as the diameter of ball utilized in planetary ball-milling process getting larger.

**Figure 5:**
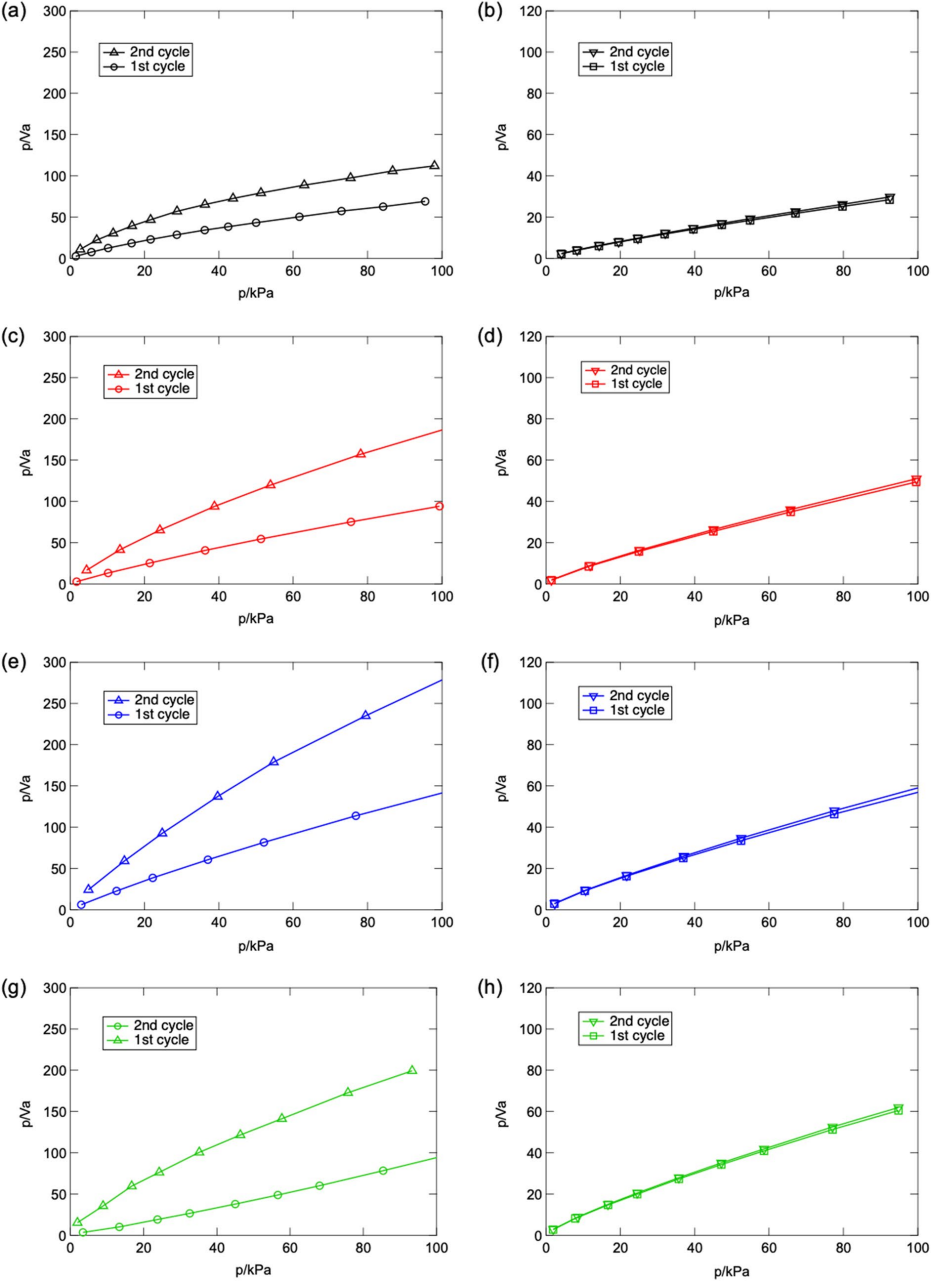
Adsorption isotherms of CO_2_/NH_3_ on (**a**, **b**) raw and mechanochemical treated HAp via (**c**, **d**) 3 mm, (**e**, **f**) 10 mm and (**g**, **h**) 15 mm under different temperatures before (1st cycle) and after (2nd cycle) in-situ vacuum treatment.

As the catalytic reaction mechanism revealed previously, the surface acidic/basic properties of HAp also play important role in deciding reaction routes and by-/final-products during oxidation of VOC^25^. Thus, the surface acidity/basicity of raw and mechanochemically treated HAp are evaluated through NH_3_/CO_2_ gas adsorption measurement and the resultant Langmuir adsorption isotherms of NH_3_/CO_2_ are shown in Fig. 5. As the corresponding results summarized in Table 1, significant enhancement of basic site population is confirmed in mechanochemically treated HAp to compare with raw powder. Regards to the above results of XRD, ATR-FT/IR, XPS and ESR characterizations, as well as detailed discussion on the chemical structure changing in mechanochemical treatment, the harvested basic sites must be attributed to the formation of surface defect in PO_4_^3−^ site of HAp. To compare HAp treated through 15 mm ball with 10 mm one, rare change of basic site population is observed. Such phenomenon can be ascribed to the increased substitution of type A/B CO_3_^2−^ and HPO_4_^2−^ formation, which suppresses the surface basicity of HAp due to the lower basicity of CO_3_^2−^ than OH^−^/PO_4_^3−^, and the acidic nature of HPO_4_^2−^ ion. Therefore, not only the chemical structure but also corresponding acidity/basicity on HAp is tuned through mechanochemical treatment.

**Table 1:** Observed adsorption amount of NH_3_/CO_2_ and estimated acidic/basic sites population on raw and mechanochemically activated HAp surface.

Sample	Adsorption amount (μmol/m^2^)		Site population (%)	
	NH_3_	CO_2_	Acidic	Basic
Raw	0.216	0.440	32.96	67.04
ϕ3 mm	0.081	0.637	11.25	88.75
ϕ10 mm	0.058	0.598	8.87	91.13
ϕ15 mm	0.090	0.972	8.41	91.59

**Figure 6:**
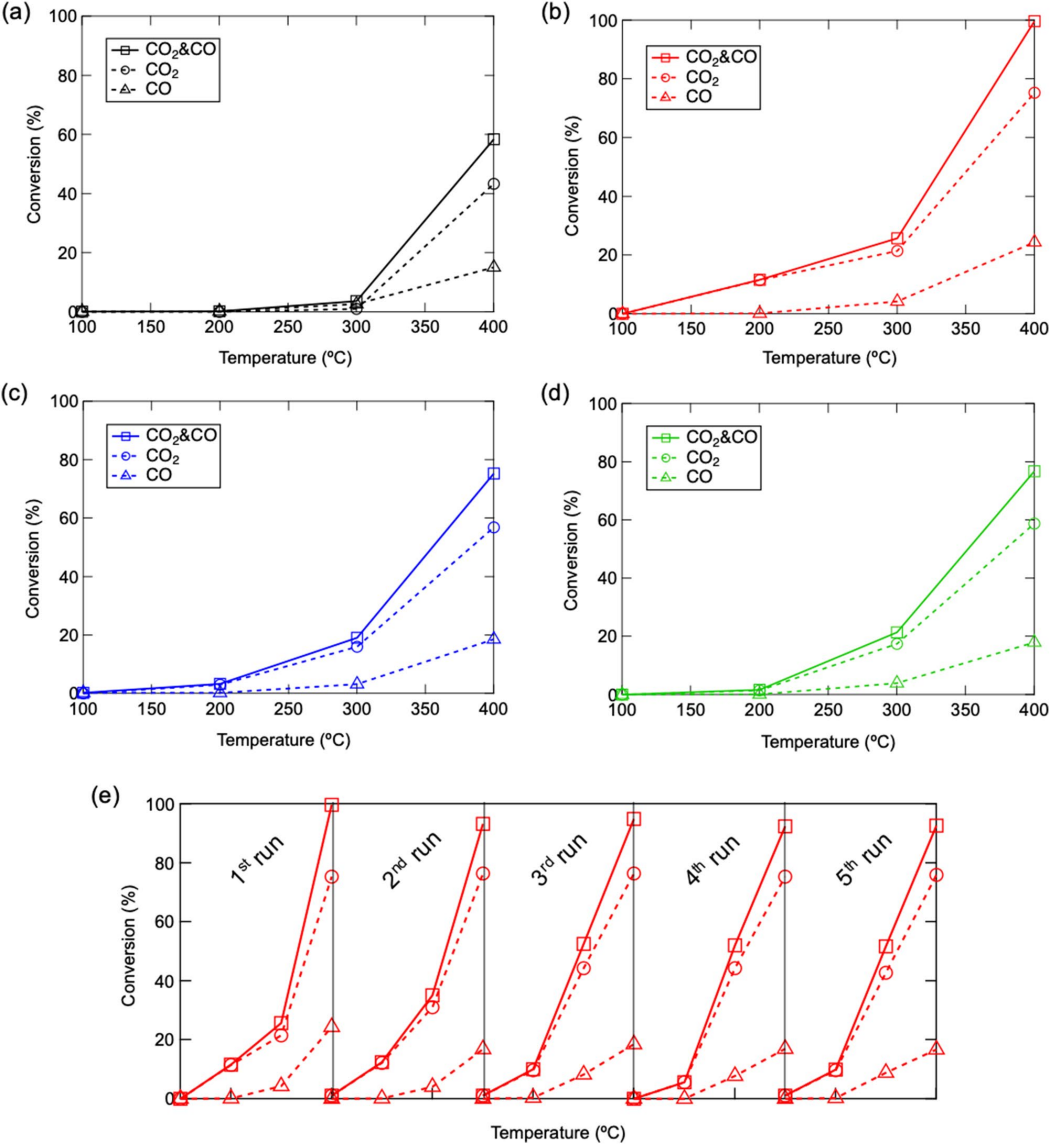
CO_2_/CO conversion of VOC under different temperatures on (**a**) raw and mechanochemically activated HAp via (**b**) 3 mm, (**c**) 10 mm and (**d**) 15 mm ball, and (**e**) cyclic test of VOC decomposition on HAp activated via 3 mm ball.

**Scheme 1:**
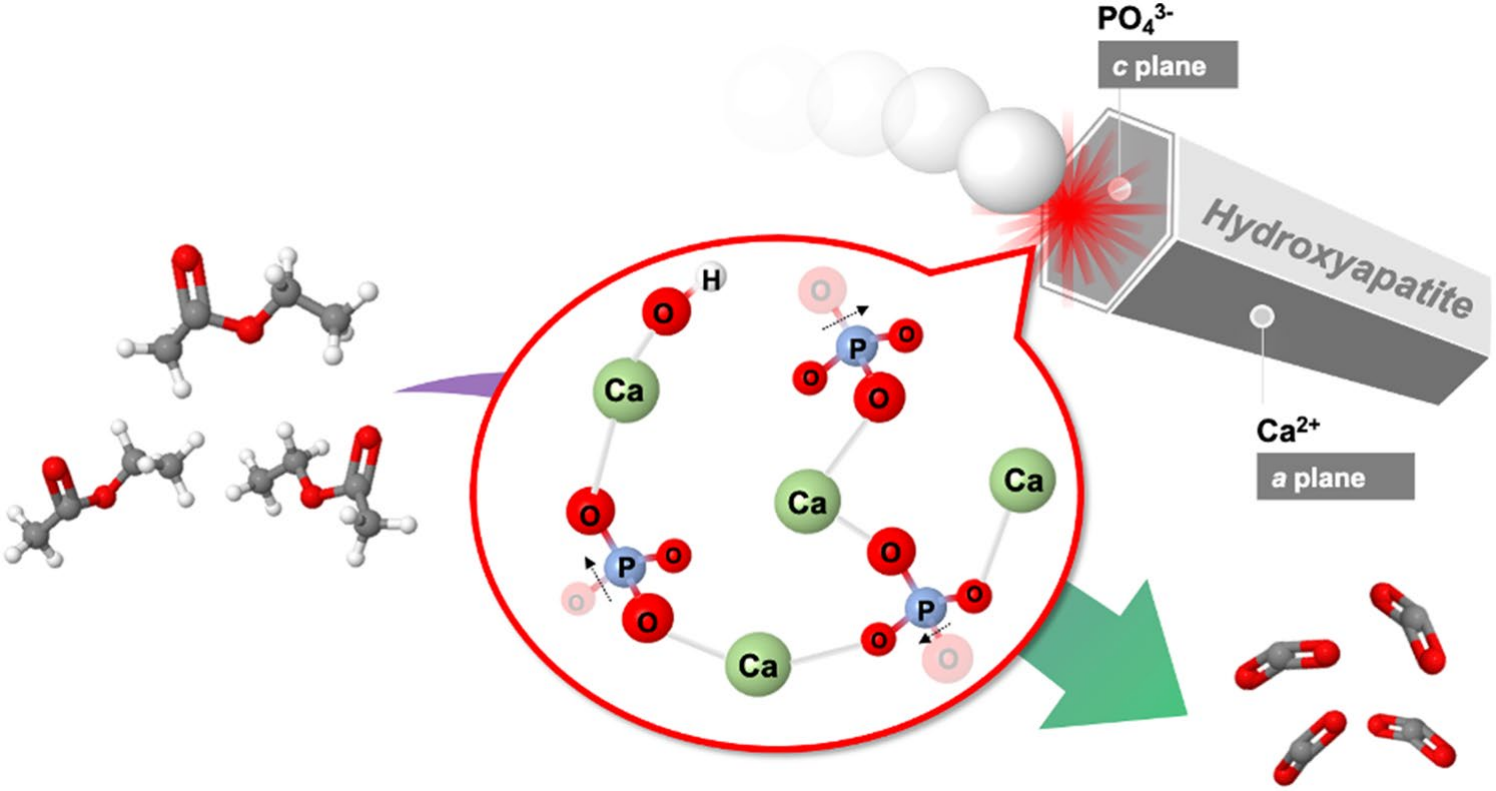
Catalytic oxidation of VOC on mechanochemical tailored new surface of HAp towards 100% CO_2_/CO conversion.

The catalytic performance of mechanochemically activated HAps is tested for oxidative decomposition of VOC by utilizing ethyl acetate as target gas. The resultant CO_2_/CO conversion of VOC at temperature range from 100 to 400 °C are displayed in Fig. 6. To compare with raw HAp which shows catalytic activity under temperature higher than 200 °C, all mechanochemically activated HAps starts to decompose VOC under lower temperature region. In addition, the catalytic performance is obviously enhanced in surface activated HAps. It worth noting that the highest conversion efficiency of 100% is successfully achieved in HAp treated by 3 mm ball. Such superior catalytic activity is steady upon cyclic tests, as result outlined in Fig. 6e. We suggest the superior catalytic performance of mechanochemically treated HAp can be attributed to the activated new surface with well-controlled chemical structure which exhibits more sufficient radical producing through defect/oxygen vacancy in PO_4_^3−^ site and an enhanced basicity. A more detailed catalytic reaction mechanism for enhanced catalytic activity and highly-efficient CO_2_/CO conversion can be described as followings. Firstly, the intensified active radicals promote the intramolecular cleavage of ethyl acetate, which results in efficient generation of ethanol and acetaldehyde. Secondly, harvested surface basic sites facilitated the following dehydrogenation of ethanol to generate acetaldehyde, which was finally oxides into CO_2_/CO efficiently by active radicals. A brief summary of above mechanism is illustrated as Scheme 1.

**Figure 7:**
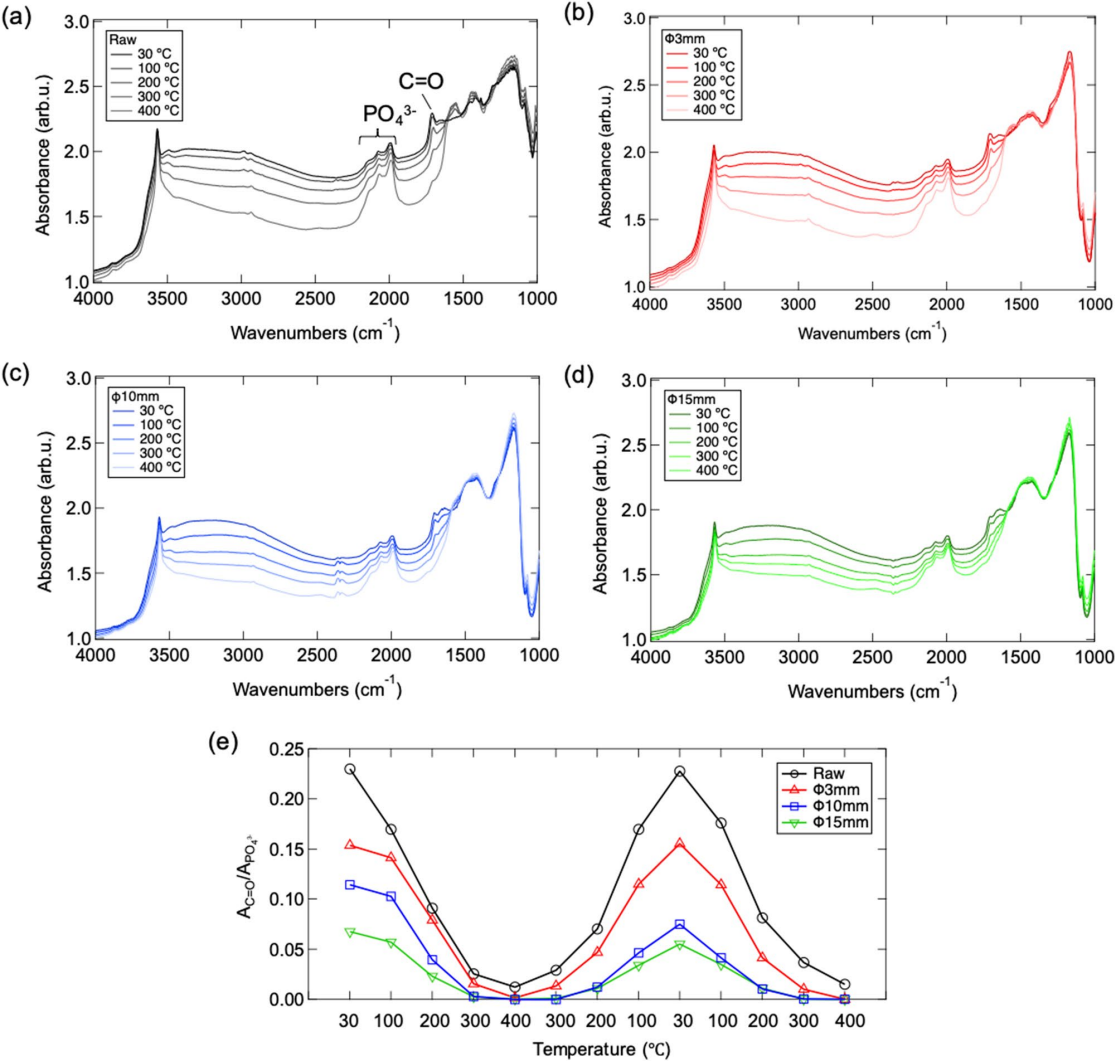
DRIFT spectra of (**a**) raw and mechanochemical treated HAp via (**b**) 3 mm, (**c**) 10 mm and (**d**) 15 mm balls under in-situ flow of ethyl acetate when temperature increasing from 30 to 400 °C and (**e**) summarized temperature-dependent integral peak area ratio of C=O/PO_4_^3−^ in cycled temperature increasing, decreasing and re-increasing processes.

Interestingly, although HAp mechanochemically treated by 10 and 15 mm exhibit better catalytic activity to compare with raw HAp, some kind of shielding effect occurs despites the extra surface defects and basicity than 3 mm case. To clarify such phenomenon, the surface adsorption property of ethyl acetate on HAp is further investigated through an in-situ DRIFT characterization^26^. As the spectra shown in Fig. 7a–d, despites the characteristic peaks of apatitic OH, overtone mode of PO_4_^3−^ and stretching vibration of CO_3_^2−^ in HAp observed at 3570, 1950 ~ 2100 and 1490 ~ 1545 cm-1, respectively, a sharp peak appears at 1750 cm^−1^ due to C=O bond originated from surface adsorbed ethyl acetate is also confirmed. By comparing the peak area ratio of C=O/PO_4_^3−^ under different temperature as summarized in Fig. 7e, it can be clearly implied that the adsorption amount of ethyl acetate reduces on mechanochemically tailored surface. Such phenomenon must be caused by the chemical structure changing of c-plane through mechanochemical stress with the fact that ethyl acetate is adsorbed on HAp surface through the bonding of carboxyl group with hydroxyl group on c-plane^20^. In addition, the adsorption amount of ethyl acetate on HAp surfaces doesn’t change in cycled temperature increasing, decreasing and re-increasing processes. Thus, we suggest that the suppressed catalytic activity in HAps treated by 10 and 15 mm ball can be attributed to the inhibited surface adsorption of ethyl acetate due to the fact that catalytic reaction occurs at the gas–solid interface cannot be established without sufficient adsorption of reactants at the active surface.

By summarizing the surface activation of HAp with respects to defect/oxygen vacancy tailoring, active radical generation, basicity/acidity and ethyl acetate adsorption tuning, it can be concluded that: (1) HAp mechanochemically treated via 3 mm ball exhibits more sufficient radical generation and possesses stronger basicity than raw material through surface defect/oxygen vacancy derived on PO_4_^3−^ site of c-plane, while the corresponding ethyl acetate adsorption is slightly suppressed since hydroxyl group existed also on c-plane was demonstrated as the adsorption center for carboxyl group of ethyl acetate. (2) Although radical production and basicity increasing was further promoted on HAps activated via mechanochemical treatment by 10 and 15 mm balls, the substitution of CO_3_^2−^ and HPO_4_^2−^ formation partially occur on both of –OH and PO_4_^3−^ sites under strong mechanical stress cannot be ignored. Adsorption property of ethyl acetate is also significantly restrained on HAps with such an excessively tailored c-plane surface. Thus, the superior catalytic performance with maximum of 100% CO_2_/CO conversion successfully achieved in HAp mechanochemically treated by 3 mm ball can be ascribed to the new surface accompanied by sufficiently generated active radical, balanced surface basicity and comparable VOC adsorption affinity.

## Conclusions

In conclusion, HAp with structurally well-controlled new surface is successfully prepared through facile one-step mechanochemical treatment in ambient air and superior catalytic activity with 100% CO_2_/CO conversion towards oxidative decomposition of VOC is achieved. Structure changes in mechanochemically treated HAp concerning particle morphology, crystallinity, chemical structure with respects to surface defect/oxygen vacancy generation in PO_4_^3−^ site, CO_3_^2−^ substitution and HPO_4_^2−^ formation, radical generation, surface acidity/basicity and VOC affinity is systemically investigated. The intensified active radicals, enhanced surface basicity and comparable VOC adsorption established in mechanochemical treated HAp facilitate the highly-efficient CO_2_/CO conversion. Regards to the cost-effectiveness and non-toxic nature of HAp, as well as the sustainable surface tuning process through mechanochemical treatment, the superior catalytic performance reported in this study opens new strategy for development of highly-efficient noble-metal-free catalyst for VOC elimination and future environmental cleaning techniques.

## Methods

### Surface activation of HAp via mechanochemical treatment

A stoichiometric HAp with sphere shape (provided by Taihei Chemical Industrial Co., Ltd.) is utilized as raw powder. Mechanochemical treatment is conducted on a commercial high-energy planetary ball-milling equipment (Premium Line 7, Fritsch Japan Co., Ltd.) in experiment. During mechanochemical treatment, 4 g of HAp powder (the surface physically adsorbed water has been removed via vacuum drying under 60 °C for 24 h) and 80 g of zirconia (ZrO_2_) ball are loaded into sample vessel under air atmosphere. The diameter of ZrO_2_ ball is varied as 3, 10, 15 mm, while the rotation speed is settled as 300 rpm and treatment time is fixed as 3 h.

### Characterizations of raw and mechanochemically treated Haps

The morphology of raw and mechanochemical treated HAp powders are observed by scanning electron microscopy (SEM: JCM-6000 NeoScope, JEOL Ltd.). The specific area of HAps is evaluated based on BET equation via a commercial nitrogen adsorption/desorption system (BELSORP miniX, MicrotracBEL Corp.). The crystallinity of HAp powders is confirmed by powder X-ray diffraction equipment (PXRD: Ultima IV, Rigaku Corp.) with a Cu Kα line during where the operating current and voltage are settled as 40 mA and 40 kV, respectively. The chemical structure of HAp is analyzed via Fourier transform infrared spectrometer equipped with attenuated total reflection unit (ATR-FT/IR: FT/IR-V6000, JASCO Corp.) and X-ray photoelectron spectroscopy (XPS: M-Probe, Surface Science Instruments). Surface defects/vacancies and generated radicals are monitored by an electron spin resonance instrument (ESR: JES-FA200, JEOL Ltd.) equipped with in-situ heating system. The surface acidity/basicity is quantified through NH_3_/CO_2_ adsorption on a commercial vapor adsorption system (BELSORP MAXII, MicrotracBEL Corp.). The surface adsorption property of VOC on HAp is investigated by a diffuse reflectance infrared Fourier transform spectroscopy (DRIFT: FT/IR-V6000, JASCO Corp.) with self-designed in-situ VOC flow chamber, the detailed construction has been described in our previous report^26^.

### Catalytic activity test on HAp towards VOC decomposition

The catalytic activity of HAp for VOC decomposition is tested based on experimental system as reported by our group previously^25,27^. In this study, ethyl acetate with concentration of 100 ppm is utilized as VOC source, which is mixed with air in a volume ratio of 1:1 at a constant flow rate both of 250 mL/min through mass flow meters. As for the decomposed VOC, the concentration of inorganic products CO_2_ and CO are detected by infrared absorption CO2 (RI-215D: RIKEN KEIKI Co., Ltd.) and CO (UM-300: KITAGAWA KOMYO RIKAGAKU KOGYO) monitors. The conversion efficiency of VOC is calculated by the following equation:

$$Conversion\% = \frac{{Dectected\;{\text{CO}}_{2} /{\text{CO}}}}{{Theoredical\;{\text{CO}}_{2} /{\text{CO}}}} \times 100 \%$$

## References

[1] Lemieux PM, Lutes CC, Santoianni DA (2004). Emissions of organic air toxics from open burning: a comprehensive review. Prog. Energy Combust. Sci..

[2] Huang Y, Ho SSH, Lu Y, Niu R, Xu L, Cao J, Lee S (2016). Removal of indoor volatile organic compounds via photocatalytic oxidation: a short review and prospect. Molecules.

[3] Placet M, Mann CO, Gibert RO, Niefer MJ (2000). Emissions of ozone precursors from stationary sources: a critical review. Atmos. Environ..

[4] Dunn RF, EI-Halwagi MM (1994). Selection of optimal VOC-condensation systems. Waste Manag..

[5] Zhang X, Gao B, Creamer AE, Cao C, Li YJ (2017). Adsorption of VOCs onto engineered carbon materials: a review. Hazard. Mater..

[6] Yoshikawa M, Zhang M, Toyota K (2017). Biodegradation of volatile organic compounds and their effects on biodegradability under co-existing conditions. Microbes Environ..

[7] Lewandowski DA (2017). Design of Thermal Oxidation Systems for Volatile Organic Compounds.

[8] Guo Y, Wen M, Ki G, An T (2021). Recent advances in VOC elimination by catalytic oxidation technology onto various nanoparticles catalysts: a critical review. Appl. Catal. B: Environ..

[9] He C, Cheng J, Zhang X, Douthwaite M, Pattisson S, Hao Z (2019). Recent advances in the catalytic oxidation of volatile organic compounds: a review based on pollutant sorts and sources. Chem. Rev..

[10] Huang H, Xu Y, Feng Q, Leung DY (2015). Low temperature catalytic oxidation of volatile organic compounds: a review. Catal. Sci. Technol..

[11] Loitta LF (2010). Catalytic oxidation of volatile organic compounds on supported noble metals. Appl. Catal. B: Environ..

[12] Song S, Zhang S, Zhang X, Verma P, Wen M (2020). Advances in catalytic oxidation of volatile organic compounds over Pd-supported catalysts: recent trends and challenges. Front. Mater..

[13] Mamaghna AH, Haghighat F, Lee C-S (2017). Photocatalytic oxidation technology for indoor environment air purification: the state-of-the-art. Appl. Catal. B: Environ..

[14] Kang I-S, Xi J, Hu H-Y (2018). Photolysis and photooxidation of typical gaseous VOCs by UV Irradiation: removal performance and mechanisms. Front. Environ. Sci. Eng..

[15] Kim HH, Ogata A, Futamura S (2007). Complete oxidation of volatile organic compounds (VOCs) using plasma-driven catalysis and oxygen plasma. Int. J. Plasma Environ. Sci. Technol..

[16] Li WB, Wang JX, Gong H (2009). Catalytic combustion of VOCs on non-noble metal catalysts. Catal. Today.

[17] Perez H, Navarro P, Delgado JJ (2011). Mn-SBA15 catalysts prepared by impregnation: influence of the manganese. Appl. Catal. A: General.

[18] Li S, Hao Q, Zhao R, Liu D, Duan H, Dou B (2016). Highly efficient catalytic removal of ethyl acetate over Ce/Zr promoted copper/ZSM-5 catalysts. Chem. Eng. J..

[19] Akram S, Wang Z, Chen L, Wang Q, Shen G, Han N, Chen Y, Ge G (2016). Low-temperature efficient degradation of ethyl acetate catalyzed by lattice-doped CeO_2_-CoO_x_ nanocomposites. Catal. Comm..

[20] Delimaris D, Ioannides T (2008). VOC oxidation over MnO_x_-CeO_2_ catalysts prepared by a combustion method. Appl. Catal. B: Environ..

[21] Pecchi G, Reyes P, Zamora R, Cadus LE, Fierro JLG (2008). Surface properties and performance for VOCs combustion of LaFe_1-y_Ni_y_O_3_ perovskite oxides. J. Solid State Chem..

[22] Lin K, Wu C, Chang J (2014). Advances in synthesis of calcium phosphate crystals with controlled size and shape. Acta Biomater..

[23] Okada M, Matsumoto T (2015). Synthesis and modification of apatite nanoparticles for use in dental and medical applications. Jpn. Dent. Sci. Rev..

[24] Ferri M, Campisi S, Gervasini A (2019). Nickel and cobalt adsorption on hydroxyapatite: a study for the de-metalation of electronic industrial wastewaters. Adsorption.

[25] Xin Y, Ando Y, Nakagawa S, Nishikawa H, Shirai T (2020). New possibility of hydroxyapatites as noble-metal-free catalysts towards complete decomposition of volatile organic compounds. Catal. Sci. Technol..

[26] Xin Y, Ikeuchi H, Hong J, Nishikawa H, Shirai T (2019). Oxidative decomposition of volatile organic compound on hydroxyapatite with oriented crystal structures. J. Ceram. Soc. Jpn..

[27] Nishikawa H, Oka T, Asai N, Simomichi H, Shirai T, Fuji M (2012). Oxidative decomposition of volatile organic compounds using thermally-excited activity of hydroxyapatite. Appl. Surf. Sci..

[28] Balaz P, Achimovicova M, Balaz M, Billik P, Cherkezova-Zheleva Z, Criado JM, Delogu F, Dutkova E, Gaffet E, Gotor FJ, Kumar R, Mitov I, Rpjac T, Senna M, Streletskii A, Wieczorek-Ciurowa K (2013). Hallmarks of mechanochemistry: from nanoparticles to technology. Chem. Soc. Rev..

[29] Raynaud S, Champion E, Bernache-Assollant D, Thomas P (2002). Calcium phosphate apatites with variable Ca/P atomic ratio I. Synthesis, characterization and thermal stability of powders. Biomaterials.

[30] Viswanath B, Ravishankar N (2008). Controlled synthesis of plate-shaped hydroxyapatite and implications for the morphology of the apatite phase in bone. Biomaterials.

[31] Fahami A, Nasiri-Tabrizi B, Ebrahimi-Kahrizsangi R (2012). Synthesis of calcium phosphate-based composite nanopowders by mechanochemical process and subsequent thermal treatment. Ceram. Int..

[32] Oh SC, Wu Y, Tran DT, Lee IC, Lei Y, Liu D (2016). Influences of cation and anion substitutions on oxidative coupling of methane over hydroxyapatite catalysts. Fuel.

[33] Beasley MM, Bartelink EJ, Taylor L, Miller RM (2014). Comparison of transmission FTIR, ATR, and DRIFT spectra: implications for assessment of bone bioapatite diagenesis. J. Archaeol. Sci..

[34] Tkalcec E, Sauer M, Nonninger R, Schmidt H (2001). Sol-gel-derived hydroxyapatite powders and coatings. J. Mater. Sci..

[35] Syed KA, Pang S-F, Zhang Y, Zhang Y-H (2013). Micro-Raman observation on the H_2_PO_4_^-^ association structures in a supersaturated droplet of potassium dihydrogen phosphate (KH_2_PO_4_). J. Chem. Phys..

[36] Elzinga EJ, Sparks DL (2007). Phosphate adsorption onto hematite: an in situ ATR-FT/IR investigation of the effects of pH and loading level on the mode of phosphate surface complexation. J. Coll. Inter. Sci..

[37] Diallo-Garcia S, Osman MB, Krafft J-M, Casale S, Thomas C, Kuno J, Costentin G (2014). Identification of surface basic sites and acid-base pairs of hydroxyapatite. J. Phys. Chem. C.

[38] Osman MB, Krafft J-M, Thomas C, Yoshioka T, Kubo J, Costentin G (2019). Importance of the nature of the active acid/base pairs of hydroxyapatite involved in the catalytic transformation of ethanol to n-butanol revealed by operando DRIFTS. Chem. Cat. Chem..

[39] Vandecandelaere N, Rey C, Drouet C (2012). Biomimetic apatite-based biomaterials: on the critical impact of synthesis and post-synthesis parameters. J. Mater. Sci.: Mater. Med..

[40] Gheisari H, Karamian E, Abdellahi M (2015). A novel hydroxyapatite –Hardystonite nanocomposite ceramic. Ceram. Int..

[41] Uskokovic V (2020). X-ray photoelectron and ion scattering spectroscopic surface analyses of amorphous and crystalline calcium phosphate nanoparticles with different chemical histories. Phys. Chem. Chem. Phys..

[42] Negrila CC, Predoi MV, Iconaru SL, Predoi D (2018). Development of zinc-doped hydroxyapatite by sol-gel method for medical applications. Molecules.

[43] Zhu H, Guo D, Zang H, Hanaor DAH, Yu S (2020). Enhancement of hydroxyapatite dissolution through krypton ion irradiations. J. Mater. Sci. Technol..

[44] Cipreste MF, Peres AM, Cotta AAC, Aragon FH, Antunes AM, Leal AS, Macedo WAA, Sousa EMB (2016). Synthesis and characterization of ^159^Gd-doped hydroxyapatite nanorods for bioapplications as theranostic systems. Mater. Chem. Phys..

[45] Chinnadurai D, Selvaraj AR, Rajendiran R, Kumar GR, Kin H-J, Viswanathan KK, Prabakar K (2018). Inhibition of redox behaviors in hierarchically structured manganese cobalt phosphate supercapacitor performance by surface trivalent cations. ACS Omega.

